# *ZNF582* hypermethylation as a highly specific biomarker for triage of high-grade cervical lesions in HR-HPV positive women

**DOI:** 10.3389/fmed.2025.1687869

**Published:** 2025-12-11

**Authors:** Yixiang Lian, Shali Jiang, Suran Jiang, Jiali Wang, Haijun Luo

**Affiliations:** Department of Pathology, The Affiliated Changsha Central Hospital, Hengyang Medical School, University of South China, Changsha, China

**Keywords:** *ZNF582* methylation, p16/Ki67, cervical screening, cervical intraepithelial neoplasia, high-risk human papillomavirus

## Abstract

**Objective:**

Persistent infection with high-risk human papillomavirus (HR-HPV) is the primary cause of cervical cancer (CC). DNA methylation is a promising biomarker for cervical cancer screening. This study aimed to validate the clinical efficacy of a cytological DNA methylation-based ZNF 582 methylation (*ZNF582^m^*) test for triage in detecting cervical intraepithelial neoplasia (CIN) grade 3 or higher (CIN3+) among women with HR-HPV infection.

**Methods:**

This case–control study examined 199 women who tested positive for HR-HPV from the colposcopy clinic. The cohort included 15 cases of CC, 37 cases of CIN3, 45 cases of CIN2, 49 cases of CIN1, and 53 normal cases. Using cervical pathology results as a reference, the performance of the *ZNF582^m^* test for detecting CIN3 + was evaluated and compared with liquid-based cytology (LBC) and P16/Ki67 double stain cytology (DSC).

**Results:**

*ZNF582^m^* test showed statistically significant differences in pathological results between CIN2 and CIN3 (*p* < 0.001). The area under the receiver operating characteristic curve (AUC) for distinguishing between <CIN3 and CIN3 + using *ZNF582^m^* test was 0.797 (*p* < 0.001). The *ZNF582^m^* test demonstrated higher specificity (88.4, 95% CI, 82.1–93.1%) compared to cytology (≥ atypical squamous cells of undetermined significance; ≥ASC-US+: 28.6, 95% CI: 21.4–36.6%) and P16/Ki67 DSC (43.5, 95% CI, 35.4–52.0%) for detecting CIN3+, establishing it as a superior triage biomarker for women with HR-HPV infection. The Kappa value between the *ZNF582^m^* test and pathology (<CIN3 vs. CIN3+) exhibited the highest consistency at 0.589 (0.461–0.716) when compared to other tests, indicating a strong alignment with pathological findings. In the combined test, the specificity of HPV16/18 OR *ZNF582^m^* test was found to be the highest among other combined tests, at 63.3% (95% CI = 54.9–71.1%).

**Conclusion:**

The *ZNF582^m^* test exhibiting superior triage performance compared to P16/Ki67 DSC and LBC (ASC-US+). The *ZNF582^m^* test enhances specificity (*p* < 0.001), thereby facilitating the accurate identification of CIN3 + lesions while reducing unnecessary colposcopies. This is particularly crucial in resource-limited settings, where optimizing the accuracy of triage for HR-HPV positive women is essential.

## 1 Introduction

Persistent infection with high-risk human papillomavirus (HR-HPV) is the established primary cause of cervical cancer (CC), leading the World Health Organization (WHO) and many countries to recommend HR-HPV testing as the primary screening method due to its high sensitivity in detecting high-grade cervical intraepithelial neoplasia (CIN) and CC (1). However, a challenge arises from the suboptimal specificity of HR-HPV testing, as most infections are cleared by the host immune system (2). This low specificity contributes to difficulties in follow-up, including unnecessary colposcopy referrals and strains on medical resources, underscoring the critical need for effective triage strategies. The current management of screen-positive women further faces challenges rooted in the traditional histopathological grading system for CIN (grades 1–3), which is inherently subjective and leads to considerable inter-observer variability and moderate reproducibility. This diagnostic uncertainty is clinically consequential, as evidenced by the high regression rate of CIN2 lesions [approximately 55% (3)] and the potential for under-diagnosis in CIN3 cases, where a significant proportion [approximately 14% (4)] are upgraded to cancer after surgical excision. While guidelines propose liquid-based cytology (LBC) and HPV16/18 genotyping for triage (5). LBC’s subjectivity and the shortage of skilled cytologists hinder its effectiveness. Therefore, developing objective and reproducible triage tools for HR-HPV-positive women is a critical priority in cervical cancer prevention.

DNA hypermethylation of tumor suppressor genes in the promoter region and the expression of P16/Ki67 immunohistochemistry are reported as significant markers for progressive cervical tumorigenesis. Beyond the expression of protein biomarkers such as P16/Ki67, DNA hypermethylation of tumor suppressor gene promoters represents a fundamental epigenetic mechanism in cervical carcinogenesis (6, 7). The P16 protein participates in cell cycle regulation and usually exerts an anti-proliferative effect, while the Ki-67 protein is a marker of cell proliferation. A prospective cohort study with a 5-year follow-up demonstrated that the P16/Ki67 dual stain cytology (DSC) offers more accurate risk discrimination compared to cytology in the triage of women who test positive for HR-HPV (8). The hypermethylation levels of the cervical-specific tumor suppressor gene correlate with the severity of CIN, reaching their peak in cases of CC (9, 10). DNA methylation testing may improve diagnostic accuracy compared to traditional methods due to its excellent performance and reproducibility (11). Numerous studies have indicated that highly specific DNA methylation can serve as an effective triage tool for women infected with HR-HPV (12, 13). DNA methylation testing offers several key advantages for triage. These DNA methylation detections demonstrated high reproducibility, effectively minimizing inter-observer variability (14). Furthermore, methylation markers exhibit considerable chemical stability in clinical specimens such as cervical scrapings, which enhances their robustness for routine testing applications (15). In contrast to cytology and P16/Ki67 staining, which primarily indicate active cellular changes that can be associated with transient HPV infection, promoter hypermethylation represents a cumulative event. This event marks a more advanced and typically irreversible stage in the process of malignant transformation. Consequently, the assessment of promoter hypermethylation inherently provides higher specificity for identifying women with genuine precancerous lesions (CIN2+). This enhanced specificity contributes to a reduction in unnecessary colposcopy referrals and helps alleviate associated patient anxiety (16, 17). *ZNF582* methylation (*ZNF582^m^*) test has been identified as a highly promising biomarker for the detection of cervical cancer and its precursor lesions (18–20). However, the use of *ZNF582^m^* test as a triage tool is currently limited to atypical squamous cells (ASC) in cytology (20) and there has been a lack of comprehensive research regarding the triage of HR-HPV-positive women.

This primary objective of the case–control study is to evaluate the diagnostic performance of *ZNF582^m^* test, cytology (ASC-US+), and P16/Ki67 DSC in triaging women with HR-HPV infection.

## 2 Methods

### 2.1 Participants

This case–control study, approved by the Medical Ethics Committee of the Affiliated Changsha Central Hospital, Hengyang Medical School, University of South China (Approval Number: 2022-S0165), recruited participants in the colposcopy clinic from June 2022 to June 2023. Participants were enrolled from colposcopy clinic in accordance with the established inclusion and exclusion criteria to ensure a relevant and consistent study. The Inclusion Criteria include: age ≥ 20 years, a positive test result for HR-HPV, and the provision of signed informed consent prior to enrollment. Participants were excluded from the study if they met any of the following criteria: individuals who were pregnant or lactating; those with a history of female genital tract cancer; individuals who had undergone surgery for cervical lesions (cancer); participants who received chemotherapy, radiotherapy, or immunotherapy within the past year; those with a history of non-surgical treatment for cervical lesions in the preceding 6 months; and cases with incomplete data, including but not limited to pathology, cytology, HR-HPV results, or P16/Ki67 DSC. To ensure diagnostic consistency, all cases were reviewed by dedicated gynecologic pathologists with over 10 years of experience. The final diagnostic outcomes (end-point) were classified according to the WHO guidelines (2014) into the following categories: cervicitis (Normal), CIN1, CIN2, CIN3, and cervical cancer (CC). The study flowchart is described in Supplementary Figure 1.

Cervical exfoliated cell samples were collected by gynecologic physicians using cervical brushes (Landing Intelligent Medicine Co., Ltd., Wuhan, China). These samples were subsequently preserved in ThinPrep PreservCyt Solution (Hologic Inc., MA, USA). The collected and preserved samples were then utilized for various diagnostic assays, including cytology, HR-HPV testing, methylation analysis, and P16/Ki-67 DSC. Methylation analysis and DSC were performed without knowledge of the clinical information and pathological results.

### 2.2 HR-HPV assay

HR-HPV genotypes were identified using the 14 High-risk HPV with 16/18 Genotyping Real-time PCR Kit (HBRT-H14; HybriBio Ltd., Guangzhou, China, China National Medical Device Certificate No. 20163401763) on an ABI 7500 Real-Time PCR System (Thermo Fisher Scientific Co., Foster City, USA). All procedures were performed according to the manufacturer’s instructions. Positivity was defined as follows: HPV16/18 (+) indicated infection with either type 16 or 18, while Other 12 types (+) indicated infection with any of the 12 other genotypes (31, 33, 35, 39, 45, 51, 52, 56, 58, 59, 66, 68).

### 2.3 Cytology test

Cervical exfoliated cells in PreservCyt Solution were automatically processed into thin-layer slides using the ThinPrep 5,000 Processor and Imaging System (Hologic Inc.) following the manufacturer’s protocols. Cervical thin-layer slides were evaluated by an experienced cytopathologist following the 2014 Bethesda System (TBS; third edition) criteria for classification. The TBS categorizes cytological morphological abnormalities into the following descriptions: atypical squamous cells (ASC), atypical squamous cells of undetermined significance (ASC-US), atypical squamous cells that cannot exclude high-grade squamous intraepithelial lesion (ASC-H), low-grade squamous intraepithelial lesions (LSIL), high-grade squamous intraepithelial lesions (HSIL), and squamous cell carcinoma (SCC); as well as atypical glandular cells (AGC), adenocarcinoma *in situ* (AIS), and adenocarcinoma (AD).

### 2.4 *ZNF582* methylation test

The genomic DNA of Exfoliated cervical cells were extract using a Nucleic Acid Extraction Kit (Hoomya, Changsha, China) according to the instructions. Genomic DNA (>200 ng) was subjected to bisulfite conversion using a DNA Methylation pre-treatment Kit (Hoomya Gene Technology Co., Ltd., Changsha, China). After the bisulfite reaction, unmethylated cytosine was converted to uracil, while methylated cytosine remained unchanged. The methylation level of *ZNF582* was assessed using the *ZNF582* Methylation Detection Kit (Hoomya Gene Technology Co., Ltd., Changsha, China) with the type II collagen gene (*Col2A1*) as an internal reference, and this analysis was performed on bisulfite-converted DNA via the LightCycler^®^480 quantitative PCR system (Roche Applied Science, Pennsburg, Germany) in accordance with the manufacturer’s instructions.

The cycling conditions are briefly described as follows: 95 °C for pre-denaturation lasting 10 min; followed by denaturation at 95 °C for 10 s, annealing at 70 °C for 15 s, and extension at 68 °C for 40 s. This cycle is repeated for a total of 50 cycles. Finally, the samples are stored at 4 °C for a duration of 30 min. A Cp value greater than 36 indicates insufficient DNA content, rendering the detection invalid. The methylation level of *ZNF582* is based on the difference in Cp values between the two genes, i.e., ΔCp = Cp*_ZNF582_* − Cp*_Col2A1_*. A lower ΔCp value is associated with higher levels of DNA methylation.

### 2.5 P16/Ki67 dual-stain cytological immunohistochemistry

P16/Ki67 dual-stain immunocytochemistry (DSC) was performed using the CINtec® PLUS assay (Roche Diagnostics, Basel, Switzerland; China NMPA certificated No. 20163400001) to detect co-expression of P16 and Ki-67 proteins in LBC samples, as previously validated for high-grade cervical intraepithelial neoplasia (CIN2+) detection. Automated LBC processing generated cell monolayers fixed in 95% ethanol. Dual immunocytochemistry was performed using validated monoclonal antibodies against P16 (clone E6H4) and Ki-67 (clone 274–11 AC3), with sequential visualization via diaminobenzidine (DAB) chromogen (brown color) and alkaline phosphatase-conjugated Fast Red (red color), followed by hematoxylin counterstaining. The findings were subsequently classified as positive, negative, or insufficient. Slides containing fewer than 5,000 squamous epithelial cells were deemed inadequate; however, if P16 and Ki67 DSC both immuno-positive cells were identified, the result was recorded as positive. Two certified physicians independently reviewed the slides without prior knowledge of the cytology and histology results. When the evaluations of the two physicians were found to be inconsistent, the chief physician conducted a comprehensive re-examination of the slides and subsequently rendered a final decision regarding the results.

### 2.6 Statistical analyses

We estimated the sample size using PASS 15 software (NCSS Statistical Software Co. LLC., Kaysville, USA). A sample of 47 from the CIN3 + group and 141 from the <CIN3 group achieved 91% power to detect a difference of 0.1500 between the area under the ROC curve (AUC) under the null hypothesis of 0.65 and an AUC under the alternative hypothesis of 0.80 using a two-sided z-test at a significance level of 0.050. Considering a 5% dropout rate, the minimum sample size for CIN3 + was 50 cases.

All data were analyzed using MedCalc Version 22 (MedCalc Software Ltd. Ostend, Belgium). Statistical significance was defined as a two-sided *p*-value < 0.05. Continuous variables were recorded as median [IQR], and categorical variables were recorded as frequency (%). Boxplots were constructed to observe the distribution of ΔCp for *ZNF582*^m^, and the Mann–Whitney test was performed to assess distribution differences between groups. The area under the receiver operating characteristic curve (ROC) was used to estimate the ability of *ZNF582^m^* to distinguish between <CIN2 vs. CIN2 + and <CIN3 vs. CIN3 + .

**Figure 1:**
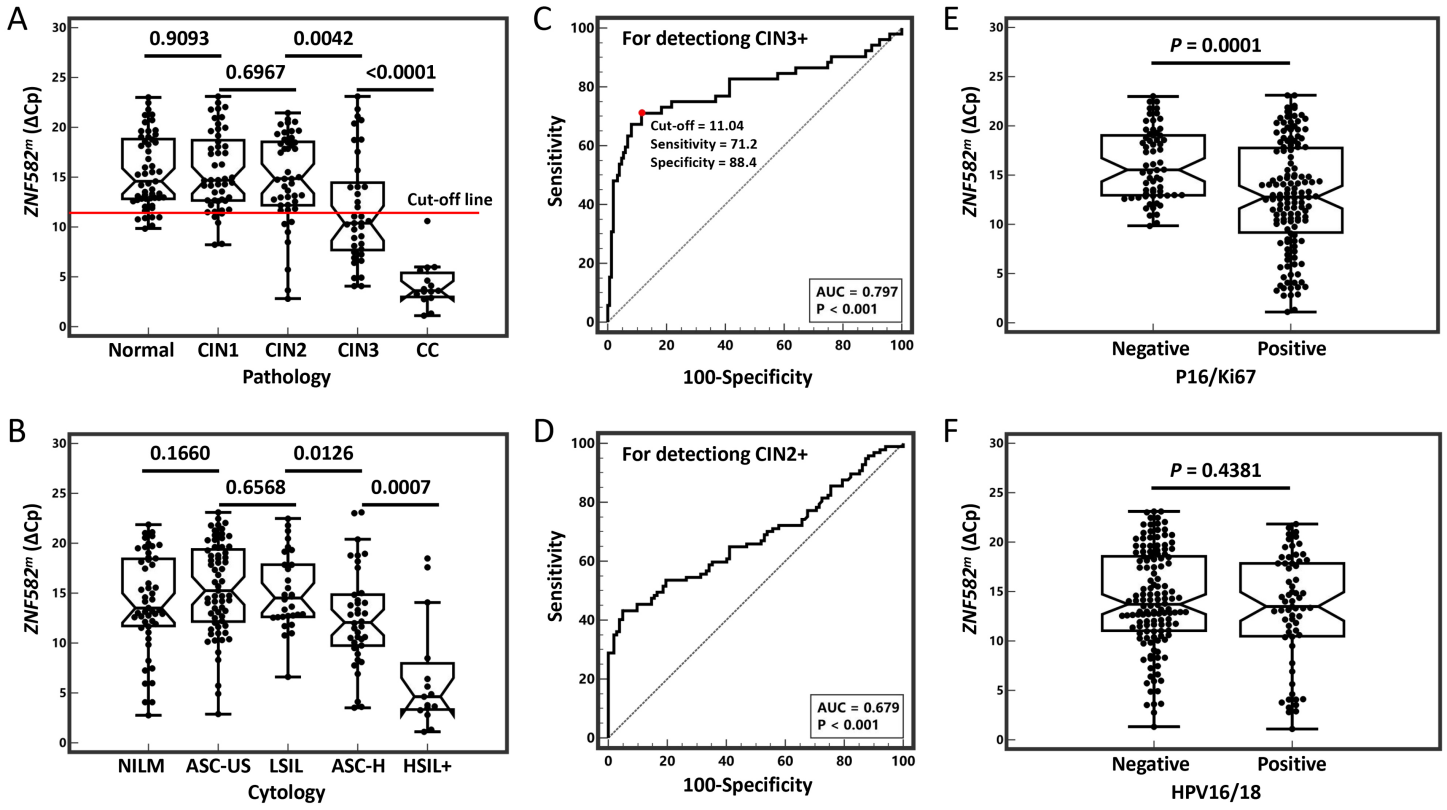
Boxplots of distribution of *ZNF582* methylation and ROC curves for detecting CIN2 + and CIN3+. **(A)** Distribution of *ZNF582^m^* in histopathologic lesions. **(B)** Distribution of *ZNF582^m^* in cytopathologic lesions. **(C)** ROC of *ZNF582^m^* for detecting CIN3+. **(D)** ROC of *ZNF582^m^* for detecting CIN2+. **(E)** Distribution of *ZNF582^m^* in P16/Ki67 results. **(F)** Distribution of *ZNF582^m^* in HPV16/18 results. CIN1, cervical intraepithelial neoplasia grade 1; CIN2, cervical intraepithelial neoplasia grade 2; CIN2+, cervical intraepithelial neoplasia grade 2 or worse; CIN3, cervical intraepithelial neoplasia grade 3; CIN3+, cervical intraepithelial neoplasia grade 3 or worse; CC, cervical cancer; NILM, no intraepithelial lesions or malignancy; ASC-US, atypical squamous cells of undetermined significance; LSIL, low-grade squamous intraepithelial lesion; ASC-H, atypical squamous cells cannot exclude HSIL; HSIL, high-grade squamous intraepithelial lesion; HR-HPV, high-risk human papillomavirus; AUC, the area under the receiver operating characteristic curve; *ZNF582^m^*, *ZNF582* methylation.

The cutoff value for *ZNF582^m^* methylation was determined by maximizing the Youden index to differentiate between histopathologically confirmed <CIN3 and CIN3+. A ΔCp value difference in crossing point between *ZNF582^m^* and *Col2A1* internal reference gene of ≤11.04 was classified as positive, whereas ΔCp > 11.04 was classified as negative (Figure 1C).

In the combined tests, “OR” indicated that any positive result is interpreted as positive, while a double-negative result was interpreted as negative; “AND,” any negative result is interpreted as negative, while a double-positive result is interpreted as positive. The agreement between test results and pathological results was estimated using inter-rater agreement (Kappa). The sensitivity and specificity for detecting CIN3 + and CIN2 + were calculated, and their 95% confidence intervals (95%CI) were estimated using the Clopper–Pearson method. The differences in sensitivity and specificity between the two triage strategies were assessed using the McNemar chi-squared test.

## 3 Results

A total of 220 participants were enrolled, among which residual cytology samples were analyzed via P16/Ki67 immunohistochemistry and residual DNA samples from HR-HPV testing were subjected to *ZNF582^m^* test. Seven DNA samples had insufficient volume for *ZNF582^m^* test, and 14 cases of P16/Ki-67 DSC tests were inadequate. Ultimately, 199 cases were included in the analysis, categorized by histopathological diagnosis: 53 with normal, 49 with CIN1, 45 with CIN2, 37 with CIN3, and 15 with CC. The median age was 42.0 years [interquartile range [IQR] 31.2–53.0]. Among the subjects, 27.1% (*n* = 54) were found to be *ZNF582^m^*-positive without any missing cases of CC, in comparison to 31.7% for participants with HPV16/18 (+) and 67.8% P16/Ki-67 DSC (+). Notably, 59.5% (22/37) of CIN3 cases and all CC cases. The prevalence of *ZNF582^m^* positivity increased with the severity of diagnosis, rising from 11.3% (6/52) in normal cases to 59.5% (22/37) in CIN3 and reaching 100% (15/15) in CC. Additional, P16/Ki67 DSC (+) is found the correlated with lesion grade, rising from 9.4% (5/53) in normal cases to 100.0% (15/15) in CC in Table 1.

**Table 1:** Characteristic of participants.

Characteristic	Normal	CIN1	CIN2	CIN3	CC	Total
*N*	53	49	45	37	15	199
Age						
Median [IQR]	43.0 [31.7–52.3]	38.0 [31.7–48.0]	35.0 [27.7–44.3]	50.0 [36.0–53.3]	60.0 [50.0–65.0]	42.0 [31.2–53.0]
HR-HPV						
Other 12 types (+), n (%)	44 (83.0)	36 (73.5)	26 (57.8)	23 (62.2)	7 (46.7)	136 (68.3)
HPV16/18 (+)	9 (17.0)	13 (26.5)	19 (42.2)	14 (37.8)	8 (53.3)	63 (31.7)
Cytology						
NILM, *n* (%)	19 (35.8)	13 (26.5)	10 (22.2)	6 (16.2)	3 (20.0)	51 (25.6)
ASC-US, *n* (%)	22 (41.5)	23 (46.9)	9 (20.0)	10 (27.0)	1 (6.7)	65 (32.7)
LSIL, n (%), *n* (%)	7 (13.2)	10 (20.4)	12 (26.7)	1 (2.7)	0 (0.0)	30 (15.1)
ASC-H, *n* (%)	3 (5.7)	3 (6.1)	10 (22.2)	18 (48.6)	4 (26.7)	38 (19.1)
HSIL+, *n* (%)	2 (3.8)	0 (0.0)	4 (8.9)	2 (5.4)	7 (46.7)	15 (7.5)
P16/Ki67						
Negative, *n* (%)	48 (90.6)	14 (28.6)	2 (4.4)	0 (0.0)	0 (0.0)	64 (32.2)
Positive, *n* (%)	5 (9.4)	35 (71.4)	43 (95.6)	37 (100.0)	15 (100.0)	135 (67.8)
*ZNF582^m^*						
Median [IQR]	14.6 [12.8–18.8]	14.7 [12.6–18.7]	14.9 [12.2–18.5]	10.4 [7.7–14.5]	3.6 [3.0–5.4]	13.6 [10.9–18.3]
*ZNF582^m^* (+), *n* (%)	6 (11.3)	4 (8.2)	7 (15.6)	22 (59.5)	15 (100.0)	54 (27.1)

CIN1, cervical intraepithelial neoplasia grade 1; CIN2, cervical intraepithelial neoplasia grade 2; CIN3, cervical intraepithelial neoplasia grade 3; CC, cervical cancer; NILM, no intraepithelial lesions or malignancy; ASC-US, atypical squamous cells of undetermined significance; LSIL, low-grade squamous intraepithelial lesion; ASC-H, atypical squamous cells cannot exclude HSIL; HSIL, high-grade squamous intraepithelial lesion; IQR, interquartile range; HR-HPV, high-risk human papillomavirus; *ZNF582^m^*, *ZNF582* methylation.

The methylation level of the *ZNF582* gene, by decreasing ΔCp values with increasing severity of cervical lesions, is strongly associated with advanced cervical lesions (CIN3+/HSIL+). The *ZNF582^m^* test showed superior diagnostic performance for CIN3 + compared to CIN2+, with a cut-off point established at 11.04 and an AUC of 0.797 for the detection of CIN3 + (Figures 1A,C). The methylation level of *ZNF582* exhibited a decrease in ΔCp with increasing cytological severity, showing significant differences for ASC-H (*p* = 0.013) and HSIL+ (*p* < 0.001) when compared to lower grades (Figure 1B). The *ZNF582^m^* test showed significant differences between positive and negative P16/Ki67 DSC results (*p* < 0.001), but no significant differences were found for HPV16/18 infection (*p* = 0.438) as shown in Figures 1E,F.

The high sensitivity of HR-HPV testing facilitates the extension of screening intervals; however, subsequent triage methods with elevated specificity are crucial for reducing colposcopy referral rates and alleviating patient anxiety. In Table 2, *ZNF582^m^* test exhibited the highest specificity (88.4% for CIN3 + and 90.2% for CIN2+), while P16/Ki-67 DSC achieved the highest sensitivity (100% for CIN3 + and 97.9% for CIN2+), respectively. In women positive for non-HPV16/18 other 12 HR-HPV types, the *ZNF582^m^* test excels with exceptional specificity. It achieved a specificity of 90.0% for detecting CIN2+, which is substantially higher than that of P16/Ki67 DSC (63.7%) and cytology (27.5%). In addition, the HPV16/18-positive group, the superior specificity of *ZNF582^m^* test was even more pronounced, reaching 90.9% for CIN2 + detection. This again far surpassed the specificity of both P16/Ki67 DSC (50.0%) and cytology (45.5%) in the same cohort. The highest specificities were observed for HPV16/18 OR *ZNF582^m^*, with values of 63.3% for CIN3 + and 70.6% for CIN2 + among the “OR” combined tests, respectively in Table 3. The combination of P16/Ki67 OR *ZNF582^m^* testing demonstrates exceptional performance by achieving perfect 100% sensitivity for detecting CIN3 + lesions while maintaining a higher specificity (40.8%) compared to other triage strategies, making it a superior approach for accurately identifying high-grade cervical disease in Table 3. The single *ZNF582^m^* test exhibited the highest specificity (90.2% for CIN2 + and 88.4% for CIN3+) compared to other single and combined tests as the triage strategies for HR-HPV-positive women, highlighting its potential as a prioritized biomarker for identifying high-grade cervical lesions with minimal false-positive.

**Table 2:** Clinical performance of single tests for detecting CIN2 + and CIN3+.

Test	AUC	Sensitivity (%) (n/N) 95%CI	Specificity (%) (n/N) 95%CI	Compared with cytology (ASC-US+)		
				*P* for AUC	*P* for sensitivity	*P* for specificity
CIN3+						
HPV16/18 (+)	0.572 (0.500–0.642)	42.3 (22/52) 28.7–56.8	72.1 (106/147) 64.1–79.2	0.745	<0.001	<0.001
Cytology (ASC-US+)	0.556 (0.484–0.627)	82.7 (43/52) 69.7–91.8	28.6 (42/147) 21.4–36.6	—	—	—
P16/Ki67 (+)	0.718 (0.650–0.779)	100 0 (52/52) 93.1–100.0	43.5 (64/147) 35.4–52.0	<0.001	0.004	0.007
*ZNF582^m^* (+)	0.798 (0.735–0.851)	71.2 (37/52) 56.9–82.9	88.4 (130/147) 82.1–93.1	<0.001	0.286	<0.001
CIN2+						
HPV16/18 (+)	0.603 (0.532–0.672)	42.3 (41/97) 32.3–52.7	78.4 (80/102) 69.2–86.0	0.336	<0.001	<0.001
Cytology (ASC-US+)	0.559 (0.487–0.629)	80.4 (78/97) 71.1–87.8	31.4 (32/102) 22.5–41.3	—	—	—
P16/Ki67 (+)	0.794 (0.731–0.848)	97.9 (95/97) 92.7–99.8	60.8 (62/102) 50.6–70.3	<0.001	<0.001	<0.001
*ZNF582^m^*	0.678 (0.608–0.742)	45.4 (44/97) (35.2–55.8)	90.2 (92/102) 82.7–95.2	0.005	<0.001	<0.001
CIN2 + in HPV16/18 (+)						
Cytology (ASC-US+)	0.630 (0.499–0.748)	80.5 (33/41) 65.1–91.2	45.5 (10/22) 24.4–67.8	—	—	—
P16/Ki67 (+)	0.738 (0.612–0.841)	97.6 (40/41) 87.1–99.9	50.0 (11/22) 28.2–71.7	0.224	0.016	0.125
*ZNF582^m^* (+)	0.662 (0.532–0.776)	41.5 (17/41) 26.3–57.9	90.9 (20/22) 70.8–98.9	0.411	<0.001	<0.001
CIN2 + in non-16/18 other 12 HR-HPV (+)						
Cytology (ASC-US+)	0.539 (0.452–0.625)	80.4 (45/56) 67.6–89.8	27.5 (22/80) 18.1–38.6	—	—	—
P16/Ki67 (+)	0.810 (0.734–0.872)	98.2 (55/56) 90.4–99.9	63.7 (51/80) 52.2–74.2	<0.001	0.004	<0.001
*ZNF582^m^* (+)	0.691 (0.606–0.767)	48.2 (27/56) 34.6–62.0	90.0 (72/80) 81.2–95.6	0.005	<0.001	<0.001

CIN2, cervical intraepithelial neoplasia grade 2; CIN3, cervical intraepithelial neoplasia grade 3; AUC, the area under the Receiver operating characteristic curve; ASC-US, atypical squamous cells of undetermined significance; CI, confidence interval; HR-HPV, high-risk human papillomavirus; *ZNF582^m^*, *ZNF582* methylation.

**Table 3:** Clinical performance of combination tests for detecting CIN2 + and CIN3 +.

Test	AUC	Sensitivity (%) (n/N) 95%CI	Specificity (%) (n/N) 95%CI	Compared with HPV16/18 OR Cytology (ASC-US+)		
				*P* for AUC	*P* for sensitivity	*P* for specificity
CIN3+						
HPV16/18 (+) OR Cytology (ASC-US+)	0.521 (0.449–0.592)	86.5 (45/52) 74.2–94.4	17.7 (26/147) 11.9–24.8	—	—	—
HPV16/18 (+) OR P16/Ki67 (+)	0.677 (0.607–0.741)	100.0 (52/52) 93.1–100.0	35.4 (52/147) 27.7–43.7	<0.001	0.016	<0.001
HPV16/18 (+) OR *ZNF582^m^* (+)	0.739 (0.673–0.799)	84.6 (44/52) 71.9–93.3	63.3 (93/147) 54.9–71.1	<0.001	>0.999	<0.001
Cytology (ASC-US+) OR P16/Ki67 (+)	0.575 (0.503–0.644)	100.0 (52/52) 93.1–100.0	15.0 (22/147) 9.6–21.8	0.047	0.016	0.424
Cytology (ASC-US+) OR *ZNF582^m^* (+)	0.623 (0.552–0.691)	98.1 (51/52) 89.7–99.9	26.5 (39/147) 19.6–34.4	<0.001	0.031	0.002
P16/Ki67 (+) OR *ZNF582^m^* (+)	0.704 (0.635–0.767)	100.0 (52/52) 93.1–100.0	40.8 (60/147) 32.8–49.2	<0.001	0.016	<0.001
P16/Ki67 (+) AND Cytology (ASC-US+)	0.699 (0.630–0.762)	82.7 (43/53) 69.7–91.8	57.1 (84/147) 48.7–65.3	<0.001	0.500	<0.001
P16/Ki67 (+) AND *ZNF582^m^* (+)	0.812 (0.750—0.863)	71.2 (37/52) 56.9–82.9	91.2 (134/147) 85.3–95.2	<0.001	0.115	<0.001
CIN2+						
HPV16/18 (+) OR Cytology (ASC-US+)	0.551 (0.479–0.622)	88.7 (86/97) 80.6–94.2	21.6 (22/102) 14.0–30.8	—	—	—
HPV16/18 OR P16/Ki67 (+)	0.745 (0.678–0.804)	99.0 (96/97) 94.4–99.9	50.0 (51/102) 39.9–60.1	<0.001	0.006	<0.001
HPV16/18 (+) OR *ZNF582^m^* (+)	0.703 (0.635–0.766)	70.1 (68/97) 59.9–79.0	70.6 (72/102) 60.7–79.2	<0.001	0.001	<0.001
Cytology (ASC-US+) OR P16/Ki67	0.598 (0.526–0.667)	99.0 (96/97) 94.4–99.9	20.6 (21/102) 13.2–29.7	0.039	0.006	>0.999
Cytology (ASC-US+) OR *ZNF582^m^* (+)	0.585 (0.514–0.655)	88.7 (86/97) 80.6–94.2	28.4 (29/102) 19.9–38.2	0.153	>0.999	0.065
P16/Ki67 (+) OR *ZNF582^m^* (+)	0.774 (0.710–0.830)	97.9 (95/97) 92.7–99.7	56.9 (58/102) 46.7–66.6	<0.001	0.023	<0.001
P16/Ki67 (+) AND Cytology (ASC-US+)	0.755 (0.689–0.813)	79.4 (77/97) 69.9–86.9	71.6 (73/102) 61.8–80.1	<0.001	0.004	<0.001
P16/Ki67 (+) AND *ZNF582^m^* (+)	0.679 (0.628–0.760)	45.4 (44/97) 35.2–55.8	94.1 (96/102) 87.6–97.8	0.008	<0.001	<0.001

CIN2, cervical intraepithelial neoplasia grade 2; CIN3, cervical intraepithelial neoplasia grade 3; AUC, the area under the Receiver operating characteristic curve; ASC-US, atypical squamous cells of undetermined significance; CI, confidence interval; HR-HPV, high-risk human papillomavirus; *ZNF582^m^*, *ZNF582* methylation.

## 4 Discussion

This case–control study presents a comprehensive validation of the DNA methylation marker *ZNF582* (*ZNF582^m^*) as a highly specific triage tool for women testing positive for HR-HPV from colposcopy clinic. Its key strength lies in its direct comparison with both established and emerging triage methods—cytology (using ASC-US+ as threshold) and P16/Ki-67 dual-stain cytology (DSC)—within the same clinical cohort. Effective triage of women with HR-HPV-positive results represents one of the most crucial clinical challenges in primary HR-HPV screening program for cervical cancer prevention. So long as cytology remains the recommended method for triage, only countries with high-quality and experienced cytologists can sustain a relatively effective HR-HPV screening approach, successfully managing the balance between detection rates and over-referral. Emerging triage methods, such as P16/Ki-67 DSC and methylation analysis, offer promising alternatives to mitigate over-referral challenges by improving specificity without compromising detection rates of CIN2+. The novel finding is that the *ZNF582^m^* test demonstrated superior specificity (88.4%) and a kappa statistic of 0.589 (95% CI: 0.461–0.716) assessing consistency with pathology for identifying CIN3+, outperforming both cytology (28.6%) and DSC (43.5%). This high specificity was achieved while maintaining a moderate sensitivity (71.2%), suggesting its primary utility is in drastically reducing unnecessary colposcopy referrals. Furthermore, the study highlights that a combined strategy of HPV16/18 genotyping OR *ZNF582^m^* testing yielded the highest specificity among all “OR” combination tests, positioning *ZNF582^m^* as a powerful, objective biomarker to optimize triage strategies in HPV positive women.

Zinc influences immune regulation and cancer prevention through its roles in oxidative stress and inflammation, while the *ZNF* family, which depends on zinc for its structure, critically regulates transcription with members acting as either oncogenes or tumor suppressors in malignant tumors (21). The integration of HR-HPV DNA into the host genome triggers epigenetic modifications (2), such as DNA methylation—an established triage tool in primary screening—which along with other changes promotes cancer development by altering gene expression and genomic stability (9, 12, 20). ZNF gene methylation is an epigenetic mechanism that silences tumor suppressor genes to promote cancer development, including CC (22) endometrial cancer (23) breast cancer (24), colorectal cancer (25, 26), and others. The *ZNF582^m^* test has demonstrated good performance in CC screening, exhibiting 45.4% sensitivity and 90.2% specificity for HSIL+, while also showing high specificity (90.9% for HPV16/18 and 90.0% for non-HPV16/18 HR-HPV) in triaging CIN2 + lesions, effectively distinguishing associated infections from non-malignant conditions.

China and 193 countries stated the WHO Global Strategy to Accelerate the Elimination of Cervical Cancer (27). The WHO, along with China (28) and numerous other countries, recommends HR-HPV testing as the primary screening method for cervical cancer (1, 5, 29, 30), which elevated positive detection rate for CIN2 + has significantly enhanced the importance of screening. However, this advancement has also introduced several challenges, including over-screening, overtreatment, and a substantial psychological burden on patients. A recognized A recognized disadvantage of HR-HPV testing is its suboptimal specificity, resulting in overtreatment and anxiety among women with transient HR-HPV infections (31). The American Society of Colposcopy and Cervical Pathology (ASCCP) recommends prioritizing the risk of CIN3 + as the primary clinical endpoint, guided by the principle of “equal management for equal risks.” This emphasis on CIN3 + is primarily due to the considerable diagnostic inconsistency associated with CIN2 classifications, which makes it a less reliable histopathologic benchmark for precancer. Furthermore, CIN2 lesions demonstrate high rates of spontaneous regression, with studies indicating that 49 to 80% may regress over 12 to 24 months without intervention, thereby reducing the urgency for immediate treatment in many cases. The recommendation is additionally supported by the significant overlap in HPV genotypes, particularly the dominance of HPV 16 and 18, observed in both CIN3 and CC, underscoring that CIN3 + represents a more advanced and consequential step in the progression toward malignancy (32). DNA methylation biomarkers are emerging as crucial tools in the clinical management of CC, particularly for refining triage strategies for HR-HPV-positive women. Accurate triage tests to identify CIN3 + are essential in primary HPV screening to prevent unnecessary physical harm, particularly in young women of reproductive age (33) Despite being a preferred screening method, cytology’s low sensitivity and operator dependence persist, and while combining it with HPV genotyping improves detection, specificity remains unenhanced, contributing to over-referral concerns (5). The single *ZNF582^m^* test exhibited high specificity as a triage tool for HR-HPV when compared to cytology (ASC-US+), achieving comparable sensitivity (*p* = 0.286) and demonstrating statistically significant differences in specificity (*p* < 0.001) for CIN3+. Furthermore, statistically significant differences were also observed in both sensitivity and specificity (*p* < 0.001) for CIN2 + (Table 2). Therefore, this study identifies the *ZNF582^m^* test as a highly specific objective triage tool that addresses the critical need of minimizing over-referral in HR-HPV primary screening by perfectly detecting all CC (100%) and demonstrating superior specificity (90.2%) for identifying CIN3 + lesions. In additional, this study showed HPV 16/18 OR *ZNF582^m^* co-test were effectively improving the specificity of detecting CIN3+, from 17.7 to 63.3% comparing to HPV 16/18 OR cytology (ASC-US+) co-test (Table 3). Furthermore, the combination of HPV16/18 genotyping with *ZNF582^m^* testing achieved the highest specificity among “OR” combined strategies, suggesting a highly efficient and cost-effective triage algorithm where HPV16/18-positive or *ZNF582^m^*-positive women are referred for colposcopy.

Previous literature studies have noted that DNA methylation as a stratification tool for HPV-positive women is equivalent to or even superior to cytology, particularly in populations that are screened based on HPV for detecting CIN3+ (34–36). The growing focus on cervical cancer methylation testing is reflected in the development of several commercial assays for screening and triage. For instance, PreCursor-M (also distributed as PreCursor-M + by Fujirebio) and QIAsure both target the methylation status of *FAM19A4* and *miR124-2*; this *FAM19A4/miR124-2* methylation test has demonstrated 77% sensitivity for CIN3, 78.3% specificity, and a negative predictive value of 99.9% for CC in HR-HPV-positive but methylation-negative cases (37). Another notable assay, GynTect (38), analyzes a six-gene panel comprising *ASTN1*, *DLX1*, *ITGA4*, *RXFP3*, *SOX17*, and *ZNF671*, showing 83.2% sensitivity and 51.6% specificity for CIN2+, outperforming both hrHPV testing and cytology alone, with further improvement in specificity when combined with hrHPV testing (39). ScreenYu Gyn^®^ specifically evaluates *ZNF671* methylation and demonstrates significantly higher sensitivity for CIN3 + compared to HPV16/18 genotyping (78.48% vs. 58.86%), while maintaining comparable specificity (67.38% vs. 69.53%) (22). Lastly, CervicalMethDx, which assesses *ZNF516*, *FKBP6*, and *INTS1* methylation, achieves 91% sensitivity, 100% specificity, and an AUC of 0.96 for CIN2+ (40). For women with positive HR-HPV screening results, *ZNF582^m^* test offered a feasible, non-invasive auxiliary diagnostic method. The low positive rates of *ZNF582^m^* test in the histopathology groups normal and CIN1 (11.3 and 8.2%) contrast with the high positive rates in CIN3 and CC (59.5 and 100.0%) (Table 1), indicating that *ZNF582^m^* test could stratify patients with abnormal cervical screening results into different management strategies. A recurring theme among these advanced methylation tests is the prominent inclusion of genes from the ZNF (Zinc Finger) family, such as *ZNF671*, *ZNF582*, and *ZNF516*. The high specificity exhibited by *ZNF582* methylation (90.2% for CIN2 + and 88.4% for CIN3+) in recent studies underscores the clinical importance of this gene family in identifying HSIL with minimal false positives. The consistent presence and strong performance of ZNF-related methylation markers across various platforms highlight their critical role in enhancing the accuracy and utility of cervical cancer screening and triage. Critically, the strong correlation between methylation levels and histological severity underscores their utility in guiding stratified management, ultimately advancing the goal of balancing screening sensitivity with specificity in cervical cancer prevention. Despite the compelling evidence from published literature demonstrating the exceptional performance of various methylation-based assays in detecting cervical pre-cancer and cancer, large-scale, multi-center clinical studies remain a crucial and necessary step for the future validation and implementation of these strategies.

Studies have shown that the simultaneous expression of P16 and Ki67 proteins in the same cell is highly correlated with persistent HR-HPV infection and the occurrence of CIN2 and CIN3 (41–43). In March 2024, the ASCCP recommended the use of P16/Ki67 DSC for triage management of HR-HPV-positive populations in HR-HPV primary screening or combined screening (44). The implementation of P16/Ki67 DSC as a triage tool for HR-HPV-positive women in Portugal’s cervical cancer organized screening programs demonstrated superior diagnostic performance compared to cytology (ASC-US+), reducing colposcopy referrals by 39.4% (45). Our previous research also showed a positive correlation between *ZNF582^m^* test and P16/Ki67 staining scores (46). The results of this study indicate that P16/Ki67 DSC showed high concordance with pathological findings (<CIN2 vs. CIN2+) (Table 4), but its specificity for detecting CIN2 + and CIN3 + is lower than that of *ZNF582^m^* test (Table 2). In women with non-16/18 HR-HPV infection, the AUC for distinguishing <CIN2 and CIN2 + using P16/Ki67 DSC was 0.810 (0.734–0.872), with a sensitivity of 98.2% and specificity of 63.7% (Table 2), both superior to cytology (all *p* < 0.010). This is consistent with previous study results (Sensitivity: 92.3%; Specificity: 51.3%) (43). Currently, P16/Ki67 DSC has been included in the guidelines as an alternative option for non-16/18 HR-HPV positive triage in China (5). The positive rate of *ZNF582^m^* test in CIN2 was relatively low, accounting for 15.6% (7/45) (Table 1). There was no statistically significant difference between CIN1 and CIN2 (*p* = 0.697, Figure 1A). Therefore, the sensitivity of *ZNF582^m^* test for detecting CIN2 + was relatively low. However, recent studies have shown that the long-term risk of CC in women with CIN2 is relatively low, with an absolute risk of 0.8–7% after 20 years (47). In addition, the high positive rate of P16/Ki67 DSC in CIN1 as a HR-HPV positive triage tool requires resampling and follow up for long time, while *ZNF582^m^* test can directly use the remaining DNA samples from HR-HPV, enabling a more efficient whole-molecular screening strategy for CC. The reflex testing, *ZNF582^m^* test, capability is a major logistical benefit, as it eliminates the need for patients to return for a separate sample collection appointment, thereby improving compliance and streamlining the clinical workflow. The core technology, real-time PCR, is already a cornerstone of modern molecular diagnostics. Most clinical laboratories engaged in HPV genotyping are already equipped with the necessary instrumentation (e.g., LightCycler^®^480, ABI 7500) and technical expertise, facilitating a relatively straightforward adoption of the *ZNF582^m^* test without major capital investment. Moreover, the high specificity of the *ZNF582^m^* test is a key driver of potential cost-effectiveness even a comprehensive economic analysis was not conducted in this study. By correctly identifying women who do not have CIN3+, it drastically reduces the number of false-positive results requiring follow-up.

**Table 4:** Concordance assessment of test outcomes with histopathological confirmation.

Test	<CIN3 vs. CIN3+		<CIN2 vs. CIN2+	
	Kappa	95% CI	Kappa	95% CI
*ZNF582^m^*	0.589	0.461–0.716	0.359	0.242–0.477
Cytology (ASC-US+)	0.071	−0.010–0.151	0.116	−0.002–0.235
Cytology (LSIL+)	0.225	0.093–0.356	0.354	0.225–0.483
HPV16/18 (+)	0.135	−0.008–0.278	0.209	0.081–0.337
P16 (+)	0.249	0.173–0.324	0.533	0.428–0.637
Ki67 (+)	0.217	0.147–0.287	0.474	0.369–0.578
P16/Ki67 (+)	0.287	0.205–0.369	0.582	0.478–0.686

CIN2, cervical intraepithelial neoplasia grade 2; CIN3, cervical intraepithelial neoplasia grade 3; ASC-US, atypical squamous cells of undetermined significance; CI, confidence interval; HR-HPV, high-risk human papillomavirus; *ZNF582^m^*, *ZNF582* methylation.

In conclusion, the *ZNF582* methylation test represents a significant advancement in cervical cancer triage. It overcomes the subjectivity and poor specificity of traditional methods by offering an objective, quantitative molecular readout that is highly correlated with high-grade disease. The limitations of this study are as follows: first, the generalizability of our findings is limited by the single-center, case–control design and the relatively small CIN3 + cohort, which did not allow for assessing the *ZNF582^m^* test’s performance in different regions or populations. Second, some HR-HPV-positive patients were unable to undergo *ZNF582^m^* and P16/Ki67 DSC, which may introduce some bias. Finally, this study did not include follow-up data, making it impossible to assess the short-term progression risks of *ZNF582^m^* test, and thus unable to recommend an accurate screening interval. Despite these limitations, this study demonstrated that the *ZNF582^m^* test in cervical exfoliated cells exhibited excellent specificity for CIN3+, surpassing traditional screening methods such as cytology and P16/Ki67 DSC for HR-HPV as primary screening method for CC. Since the combination of HPV16/18 and *ZNF582^m^* detection offers the best specificity, the finding of *ZNF582^m^* test holds promise as a replacement for cytology in the triage of HR-HPV-positive women.

## 5 Conclusion

The *ZNF582^m^* test demonstrated high clinical specificity for the detection of CIN3 + in women with HR-HPV infection, utilizing residual exfoliated cell specimens obtained from LBC or HR-HPV detection. As an objective triage biomarker, it demonstrated superior performance compared to P16/Ki67 DSC, cytology, and HPV16/18 genotyping in the triaging of women with HR-HPV positive status. It shows promise as a highly specific supplementary or alternative tool for HR-HPV triage. However, its efficacy must be evaluated in a multi-center prospective clinical trial prior to any clinical implementation.

## Data Availability

The raw data supporting the conclusions of this article will be made available by the authors, without undue reservation.
