# Supplementary material for: ZNF582 hypermethylation as a highly specific biomarker for triage of high-grade cervical lesions in HR-HPV positive women
Source: Front Med (Lausanne). 2025 Dec 11;12:1687869. doi: 10.3389/fmed.2025.1687869 (PMC12738823; doi:10.3389/fmed.2025.1687869)
Supplement: Supplementary file 1 [file Data_Sheet_1.pdf]

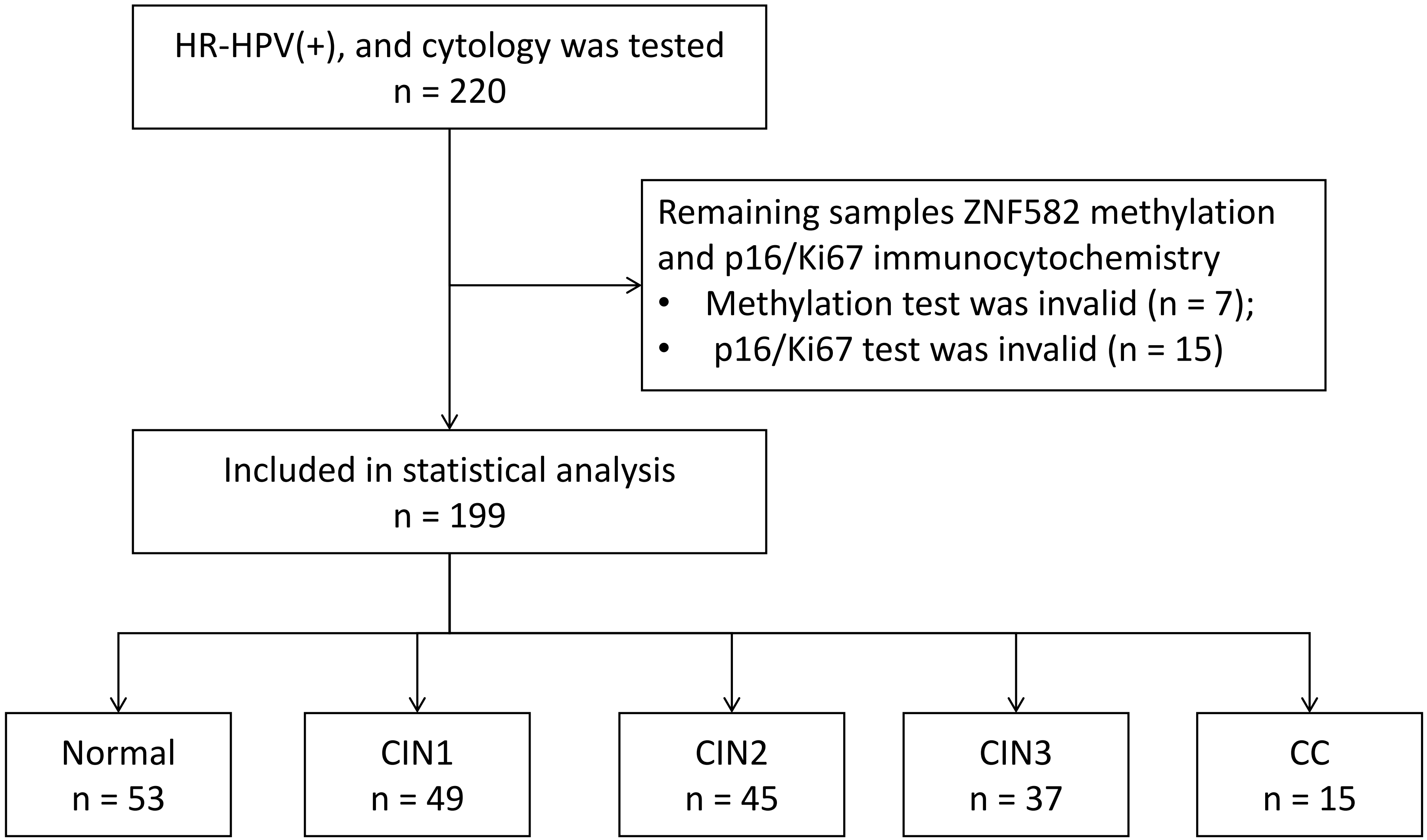

Supplementary Figure 1 Rearch flow chart.

CIN1, Cervical intraepithelial neoplasia grade 1; CIN2, Cervical intraepithelial neoplasia grade 2; CIN3, Cervical intraepithelial neoplasia grade 3;CC, Cervical cancer
